# A High Burden Human Immunodeficiency Virus and Tuberculosis Resource Limited Setting, Gains from Including Xpert MTB/RIF in the Diagnostic Algorithm of Fluid Specimens Submitted for Exclusion of Lymphoma by Immunophenotypic Analysis

**DOI:** 10.1371/journal.pone.0134404

**Published:** 2015-08-17

**Authors:** Kim Michelle Kilfoil, Elizabeth Mayne, Lesley Scott, Wendy Stevens

**Affiliations:** 1 Department of Molecular Medicine and Haematology, School of Pathology, Faculty of Health Science, University of the Witwatersrand, Johannesburg, South Africa; 2 National Health Laboratory Service, National Priority Program, Johannesburg, South Africa; University of Minnesota, UNITED STATES

## Abstract

This study investigated the benefit of incorporating Xpert MTB/RIF into the diagnostic algorithm of fluid specimens received for immunophenotypic analysis to exclude lymphoma. It was found that in a high burden HIV/TB setting, like South Africa, 130/229 (57%) of fluid specimens referred for immunophenotypic analysis to exclude lymphoma are not referred for concurrent MTBC liquid culture testing by the treating clinician. Of 99/229 (43%) specimens with corresponding culture results, Xpert sensitivity and specificity were 50% (CI:26–75%) and 99% (CI:91–100%) respectively. This demonstrates that incorporation of Xpert into the laboratory diagnostic algorithm in the immunophenotypic laboratory would improve patient work-up and care.

## Introduction

The Xpert MTB/RIF assay (Xpert) (Cepheid, Sunnyvale, CA) [1–8] is currently endorsed by the World Health Organisation (WHO) for the diagnosis of pulmonary Mycobacterial Tuberculosis Complex (MTBC) infection in sputum samples [9]. A systematic review of the Xpert for the diagnosis of pulmonary MTBC showed a pooled sensitivity of 88% and a pooled specificity of 98% [10]. The Xpert has proved to substantially reduce the diagnostic delay for pulmonary MTBC [8, 11, 12] with the added advantage of real time Rifampicin resistance testing. Resistance to Rifampicin alone is rare and more than 90% of Rifampicin resistant MTBC cases also show resistance to Isoniazid. The detection of Rifampicin resistance is therefore used a surrogate marker to detect MDRTB [11]. There is comparatively less data on the use of Xpert for the diagnosis of extra-pulmonary MTBC (EPTB) owing to variations in study population, sample size, methodology and specimen type in studies performed but is being investigated for inclusion in national Tuberculosis programs [13, 14]. The WHO meta-analysis of 15 studies showed a low pooled sensitivity of 43.7% (95% Confidence interval (CI): 24.8–64.7%) and a high pooled specificity of 98.1% (CI: 95.3–99.2%) [14]. These studies also showed that Xpert performed on fluid specimens (pleural and ascitic) have a lower sensitivity compared to other EPTB specimen types e.g. lymph nodes [1, 3, 4, 10, 12, 15–21]. The lower sensitivity may be attributable to the low bacillary load and/or the presence of an inhibitor within the fluid [22]. The major advantage of using Xpert in the diagnosis of MTBC on fluid specimens, however, is that Xpert is able to produce a result despite the high rate of contamination of extrapulmonary fluid specimens [12].

In South Africa, MTBC infection is highly prevalent with up to 70% of presumptive MTBC cases being HIV co-infected [23]. In South Africa, the incidence rate of MTBC infection increased from 2010 to 2012 from an estimated 981/100 000 population to 1000/100 000 population. In 2010, there was an approximate 60–61% HIV prevalence in incident MTBC cases which increased to 62–63% in 2012 [24, 25]. Early detection of EPTB in this high burden HIV setting is critical to improve patient care [2].

Furthermore, in HIV infection, the incidence of all subtypes of Non-Hodgkin lymphoma (NHL) is increased by 60–200 times [26, 27] with pleural effusions occurring in 20–30% of these patients [28]. In the South African setting, many fluid specimens (pleural and ascitic) received for immunophenotypic analysis by flow cytometry are from HIV positive patients suspected of lymphoma. During the diagnostic work-up of these patients, EPTB would need to be excluded. This study, therefore, investigated the role of Xpert in the laboratory diagnostic algorithm (LDA) of these fluid specimens referred for lymphoma testing. Incorporation of Xpert into the LDA would enable early identification of EPTB, identification of Rifampicin resistant MTBC, timely initiation of treatment and overall improving patient care. As the Xpert instrument is not limited to a Biosafety Level (BSL) 3 laboratory, this would allow integration of laboratory services, not limiting MTBC testing to specific areas of the laboratory.

## Materials and Methods

Ethical approval was obtained from the University of the Witwatersrand Human Research Ethics committee (HREC M121010) for this study. The committee allows for the use of residual specimen in the laboratory for validation of new technology. This can be performed without obtaining consent from the patient, with permission from the committee. This project’s protocol was submitted to the HREC committee and they waived the need for informed consent. This study took place between August 2012 and February 2013. To investigate this integration, all pleural and ascitic fluid specimens received in the immunophenotyping facility at the National Health Laboratory Services in Johannesburg were processed for routine flow cytometric analysis. A standard panel for exclusion of B and T-cell lymphoproliferative disorders and non-haemopoietic tumours (anti-kappa, anti-lambda, CD19, CD5, CD4, CD3, CD8, and CD45) was routinely performed. Any specimen with a raw (unprocessed) residual volume (>0.5ml) was then tested with Xpert according to manufacturer’s instructions for pulmonary samples. The specimens were not centrifuged, and were incubated at room temperature for 15 minutes with solvent reagent buffer in a 3:1 (0.5ml residual) or 2:1 (>0.5ml residual) ratio. Sensitivity and specificity, including 95% CI, were calculated on specimens where both liquid culture MGIT (used as the reference) and Xpert results were reported and retrieved from the Laboratory information system (LIS) [29]. Specimens without a corresponding culture result on the LIS system were not included in the statistical analysis of this study. Concurrent disease status of these patients, specifically HIV status, was not known to the authors as this information fell outside of the scope of our ethics approval.

## Results and Discussion

A total of 392 fluid specimens were received for immunophenotyping during the study period (see Table 1), of which 58.4% (229/392) had residual volume for Xpert testing. Of these, 74% (169/229) were pleural fluid and 26% (60/229) ascitic fluid. Lymphoma was not diagnosed immunophenotypically on any of the specimens tested on Xpert during the study period. Surprisingly, only 43% (99/229) of the specimens were submitted for concurrent MTBC culture by the treating clinician at any point during admission of these patients, based on the LIS history. Of these specimens, 3% (3/99) were MGIT contaminated, however a result was generated on these samples when tested on Xpert. Xpert yielded 9% (9/99) positivity compared to 17% (17/99) positive on MGIT. One false positive was detected on Xpert. MTBC positivity determined by Xpert in the non-culture specimens was 8.5% (11/130), with 1 Rifampicin resistant case identified. These cases would have remained undiagnosed for EPTB without Xpert testing in the LDA. The Xpert error rate was 7.4% (17/229). The majority of these errors were attributed to manufacturer’s error (code 5006/5007), but there was insufficient volume for repeat Xpert testing.

**Table 1:** Xpert assay performance.

**Specimens received**					
Fluid specimens received in immunophenotyping laboratory for testing					392
Specimens with sufficient residual for Xpert testing, n (%)					229 (58,4)
Pleural fluid, n (%)					169 (74)
Ascitic fluid, n (%)					60 (26)
**Specimens submitted for liquid culture (MGIT), n (%)**					**99 (43)**
	**MGIT culture**	**Xpert**	**Sensitivity**	**Specificity**	
Positive, n (%)	17 (17)	9 (9)	**(Culture reference)**	**(Culture reference)**	
Negative, n (%)	79 (79)	81 (81)	50% (26,75)	98.5% (91,100)	
Contaminated, n (%)	3 (3)				
Error, n (%)		9 (9)			
**Specimens not submitted for MTBC culture, but tested with Xpert MTB/RIF, n (%)**					**130 (57)**
		**Xpert**			
Positive, n (%)		11 (8.5)			
Negative, n (%)		111 (85)			
Error, n (%)		8 (6)			

The sensitivity of Xpert compared to MGIT is 50% (CI: 26–75%) and the specificity is 98.5% (91–100%). This is comparable to other studies performed on fluid specimens showing overlapping CIs and the abovementioned WHO meta-analysis [1, 3, 4, 11–20]. The positive predictive value of Xpert in fluid specimens from patients being investigated for lymphoma and/or TB was found to be 88.9% (51, 99) with a positive likelihood ratio of 35.5%. This indicates that a “MTBC detected” result generated by Xpert indicated a high likelihood of EPTB infection in this patient group [30].

Our study demonstrates that, in a high burden HIV/TB setting like South Africa, >50% of fluid specimens referred for immunophenotyping are not referred for MTBC culture testing by the treating clinician. Specimens that are referred for culture can take up to 6 weeks for a result, are prone to contamination and result in unnecessary diagnostic delay. This study shows that incorporating Xpert into the LDA would have the following benefits (1) access to onsite rapid testing for MTBC; (2) allow a rapid “turn-around-time” of results including Rifampicin resistance; (3) improve the diagnostic approach to HIV positive patients with effusions by increasing the number of patients tested for MTBC. In view of the fact that Xpert will be an additional test in the laboratory diagnostic algorithm, this will have financial implications and a full costing analysis would be required before implementation and policy uptake.

There is potential for Xpert to be included in the LDA in many areas of the laboratory to improve MTBC diagnosis and patient management.
